# Epilepsy Caused by an Abnormal Alternative Splicing with Dosage Effect of the SV2A Gene in a Chicken Model

**DOI:** 10.1371/journal.pone.0026932

**Published:** 2011-10-27

**Authors:** Marine Douaud, Katia Feve, Fabienne Pituello, David Gourichon, Simon Boitard, Eric Leguern, Gérard Coquerelle, Agathe Vieaud, Cesira Batini, Robert Naquet, Alain Vignal, Michèle Tixier-Boichard, Frédérique Pitel

**Affiliations:** 1 INRA-ENVT, Laboratoire de Génétique Cellulaire, Castanet-Tolosan, France; 2 CNRS-Université Toulouse III, Centre de Biologie du Développement, Toulouse, France; 3 INRA PEAT, Pôle d'Expérimentation Avicole de Tours, Nouzilly, France; 4 INSERM, Neurogénétique Moléculaire et Cellulaire, Paris, France; 5 INRA, Génétique Animale et Biologie Intégrative, Jouy-en-Josas, France; 6 CNRS, Laboratoire de Génétique Moléculaire de la Neurotransmission et des Processus Neurodégénératifs, Paris, France; 7 CNRS, Institut de Neurobiologie Alfred Fessard, Gif-sur-Yvette, France; Instituto de Ciencia de Materiales de Madrid - Instituto de Biomedicina de Valencia, Spain

## Abstract

Photosensitive reflex epilepsy is caused by the combination of an individual's enhanced sensitivity with relevant light stimuli, such as stroboscopic lights or video games. This is the most common reflex epilepsy in humans; it is characterized by the photoparoxysmal response, which is an abnormal electroencephalographic reaction, and seizures triggered by intermittent light stimulation. Here, by using genetic mapping, sequencing and functional analyses, we report that a mutation in the acceptor site of the second intron of *SV2A* (the gene encoding synaptic vesicle glycoprotein 2A) is causing photosensitive reflex epilepsy in a unique vertebrate model, the Fepi chicken strain, a spontaneous model where the neurological disorder is inherited as an autosomal recessive mutation. This mutation causes an aberrant splicing event and significantly reduces the level of *SV2A* mRNA in homozygous carriers. Levetiracetam, a second generation antiepileptic drug, is known to bind SV2A, and *SV2A* knock-out mice develop seizures soon after birth and usually die within three weeks. The Fepi chicken survives to adulthood and responds to levetiracetam, suggesting that the low-level expression of *SV2A* in these animals is sufficient to allow survival, but does not protect against seizures. Thus, the Fepi chicken model shows that the role of the SV2A pathway in the brain is conserved between birds and mammals, in spite of a large phylogenetic distance. The Fepi model appears particularly useful for further studies of physiopathology of reflex epilepsy, in comparison with induced models of epilepsy in rodents. Consequently, *SV2A* is a very attractive candidate gene for analysis in the context of both mono- and polygenic generalized epilepsies in humans.

## Introduction

Genetic reflex epilepsy (GRE), which was first described by Morgan and Morgan (1939) [1], is a type of idiopathic epilepsy in which a stimulus of any sensory modality evokes paroxysmal manifestations only in genetically predisposed subjects. This may occur in humans and animals: the epileptic manifestations are similar among various species, and range from a simple paroxysmal electrical discharge to generalized seizures (see [2]). Photosensitive epilepsy is the most common reflex epilepsy in humans; it occurs in 1 per 4000 individuals, with a higher incidence between 7 and 19 years of age [3]. Several studies have strongly supported the notion that there is a genetic etiology for photosensitive epilepsy, but no causative gene or mutation has yet been identified [4]–[7]. Two genetic animal models of photosensitive epilepsy have been extensively studied (see [2]): one is the primate, *Papio papio* [8], and the other is the Fepi strain of the Fayoumi chicken [9].

The Fepi chicken was shown to be a reliable model of the corresponding human disease [10], [11]. It carries a recessive autosomal mutation, *epi*, which affects homozygous individuals with both photosensitive and audiogenic reflex epilepsies. The generated seizures consist of stimulus-locked motor symptoms (myoclonus) followed by generalized, self-sustaining convulsions. Electroencephalographic (EEG) recordings normally show spikes and spike and waves at rest, but these patterns are suppressed during seizures and are instead replaced by desynchronized activity patterns [12]. Neurons of the prosencephalon show burst discharges at rest while those of the mesencephalon show bursts during seizures, suggesting that each of these brain areas is responsible for an intrinsic dysfunction [13]. Other investigations, including studies involving the construction and analysis of chicken embryonic brain chimeras support these conclusions [11], [14]–[16].

Here, we report the mapping of the *epi* mutation on a chicken microchromosome. In this region, the *SV2A* gene, which encodes a multifunctional, non-ion-channel protein, was found to harbor a nucleotide substitution. This substitution, suggested to be the causative mutation, leads to aberrant splicing of the *SV2A* gene, and is responsible for a dosage effect explaining the phenotype observed in the Fepi strain.

## Results and Discussion

### The genomic scan and initial mapped interval

**Figure 1:**
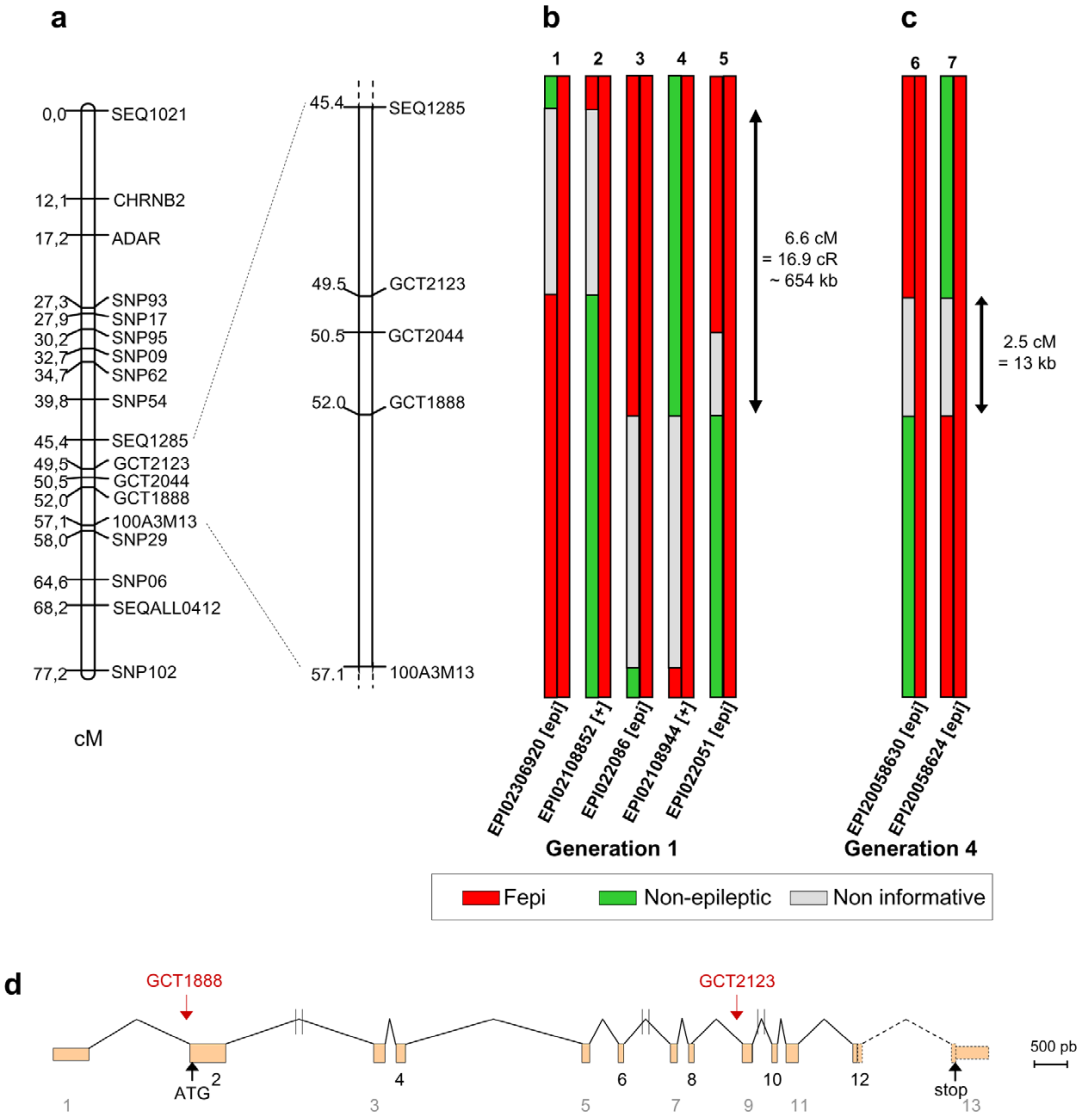
Fine mapping of the mutation that causes epileptic seizures in the Fepi chicken. (a) Genetic map of GGA25. (b) Haplotype analysis of animals from generation 1, i.e., the offspring of *epi/epi* dams crossed with carrier sires. The colors represent the origins of the chromosome haplotypes, as determined by SNP genotyping: red, epileptic line (Fepi); green, other line; gray, unknown origin (non-informative markers). An initial interval of 11.6 cM was defined between markers SEQ1285 (individuals 1 and 2) and 100A3M13 (individuals 3 and 4). Individual 5 alone restricted the interval to a 6.6-cM region having an estimated size of 650 kb based on radiation hybrid mapping data [17]. (c) Two recombinant animals obtained in the fourth generation restricted the interval to 0.5 cM (two-point genetic distance calculation based on the pedigree data). (d) This distance corresponded to 13 kb on the sequence assembly; the linked region fell between exons 2 and 9 of the *SV2A* gene. Exons 12 and 13 and intron 12 (dotted lines) were not sequenced. The vertical double lines indicate introns that were only partially sequenced.

To gain new insight into the molecular mechanisms underlying photosensitive epilepsy, we sought to identify the mutation responsible for the photosensitive epilepsy in the Fepi chicken. We performed a genome-wide linkage analysis on the first generation of a dedicated pedigree (Fig. S1). Our first genome scan, which used all available microsatellite markers found to be informative in our mapping population, excluded the known genetic map and the first generation of the chicken genome sequence assembly (February 2004) when we used a recessive model for the *epi* mutation. However, this first version of the chicken genome sequence lacked information for 10 microchromosomes, and the genetic map was incomplete. Subsequently, our work on completing the chicken genome sequence enabled us to develop new SNP markers. Genotyping of our populations using these markers allowed us to find the first evidence of linkage between the *epi* mutation and marker SEQ1009, mapped to linkage group E26C13. This led us to identify microchromosome GGA25, and develop RH (Radiation Hybrid) and genetic maps for this microchromosome [17], that was largely under-represented in the sequence assembly (Fig. 1, Fig. S2). The addition of more markers allowed us to identify an initial 11.6-cM linked genetic region falling between markers SEQ1285 and 100A3M13 (Fig. 1). However, despite the inclusion of GGA25 in the second chicken genome assembly (May 2006), very little sequence information was available; only about 1.5 Mb of gapped sequence was available for this chromosome, which has an estimated size of 11.4 Mb [17].

### Candidate genes and the refined interval

Comparative mapping suggested that this region was syntenic with human chromosome HSA1q21.1-21.2 [17], and additional markers were developed from chicken chrUn_random sequences (sequence contigs that could not be placed on a specific chromosome with any degree of confidence) showing similarities to this region of the human genome. Linkage analysis with these additional markers narrowed the interval to a 6.6-cM region for which relatively few genomic sequences were available from the chicken assembly (Fig. 1b, Fig. S2, S3, S4). In this region, a single gene, *SV2A* (synaptic vesicle glycoprotein 2A), appeared to be a very strong candidate for the *epi* mutation based on its potential involvement in neurotransmission [18] (Fig. S3). Because most of the *SV2A* gene sequence was not found in the chicken sequence assembly (http://genome.ucsc.edu/cgi-bin/hgGateway) nor among the published chicken genes or EST (http://www.ncbi.nlm.nih.gov/), we aligned chicken chrUn_random sequences to those from other model organisms and identified new genomic chicken *SV2A* fragments homologous to the mouse sequence (Fig. S4). Combining this with partial cloning and sequencing of chicken *SV2A* provided new SNP markers that were used to narrow the genetic mapping interval to within a 13-kb portion of *SV2A* in the fourth generation of our pedigree, and determined that the causative mutation laid between markers GCT1888 and GCT2123 (Fig. 1b and 1c).

### Identification of the mutation by sequencing

**Figure 2:**
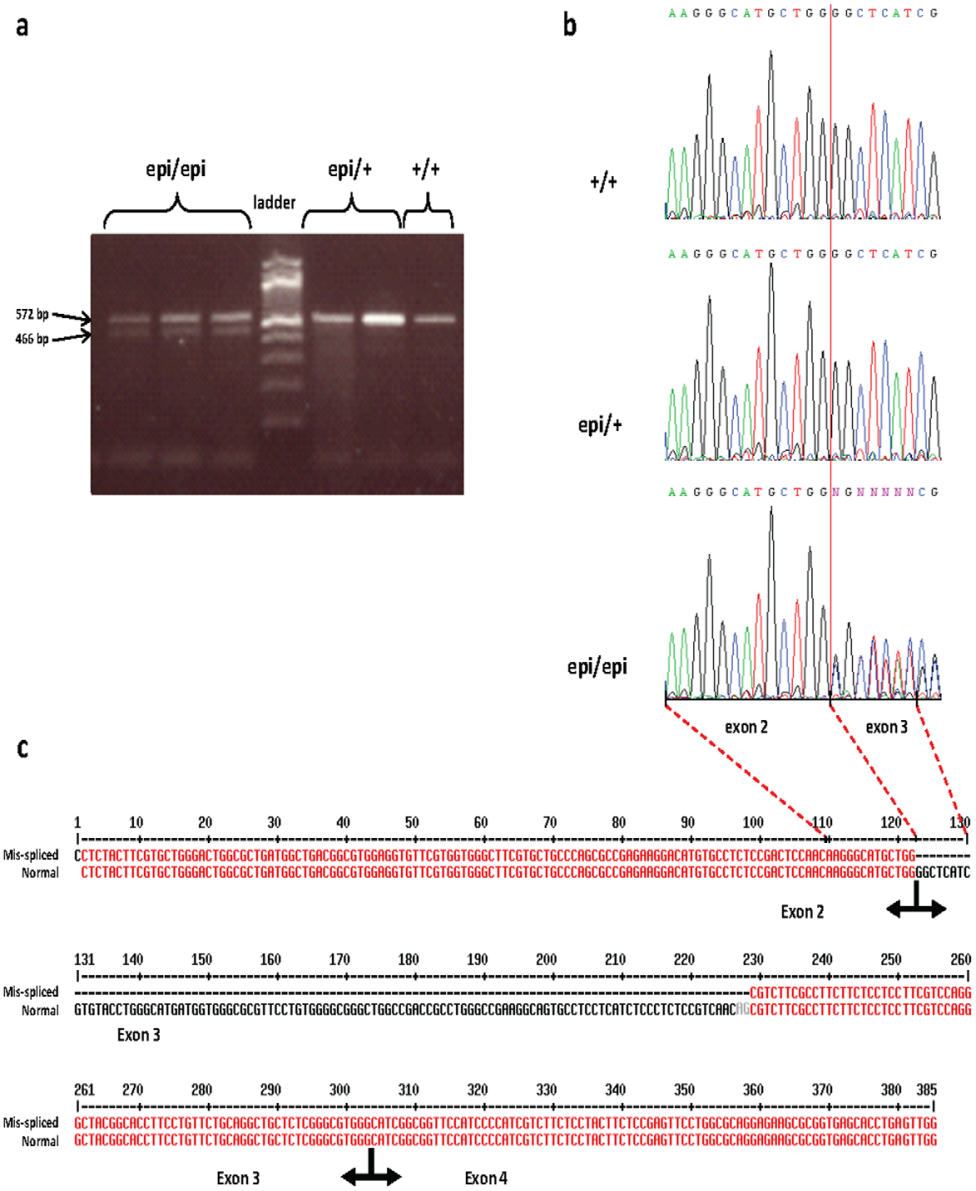
Identification of an abnormal splicing event in the *SV2A* cDNA of the Fepi chicken. (a) RT-PCR of *SV2A* in wild-type, carriers and epi/epi individuals using primers GCT1964U and GCT2146L showed the presence of an additional, smaller band in individuals homozygous for the epi mutation. (b) Direct sequencing of the PCR products (using primers GCT1964Lrev and GCT2044L) from animals of the three genotypes showed identical sequences up to the end of exon 2. Thereafter, a mixture of sequences was observed only in the homozygous *epi/epi* individuals. (c) Separate sequencing of the two bands confirmed that both corresponded to the *SV2A* cDNA, but the shorter fragment had a 106-bp deletion starting at the beginning of exon 3. The presence of an (AG) dinucleotide (red highlight) at the 3′ end of the deleted fragment suggested the possibility of a mis-splicing event.

To examine possible polymorphisms within the coding sequence of *SV2A*, we sequenced cDNA from the brains of adult epileptic animals (i.e., homozygotes), heterozygous carriers, and wild-type chickens using four overlapping primer pairs (see Methods). No polymorphism was detected, but we found that the SV2A transcripts of epileptic chickens showed abnormal alternative splicing events in exon 3 leading to the presence of an abnormal splice variant in epi/epi animals (Fig. 2). Sequencing of PCR products showed that the first 106 base pairs (bp) of *SV2A* exon 3 were missing in the alternatively spliced variant (Fig. 2); this caused a frameshift that introduced a premature termination codon 75 bp before the junction of exons 4 and 5. This could result in decreased levels of functional SV2A protein, either due to the synthesis of a truncated protein of 244 amino acids (versus 742 amino acids in the wild-type mammalian protein [19]), or degradation of the alternative mRNA via the nonsense-mediated pathway of mRNA decay [20].

**Figure 3:**
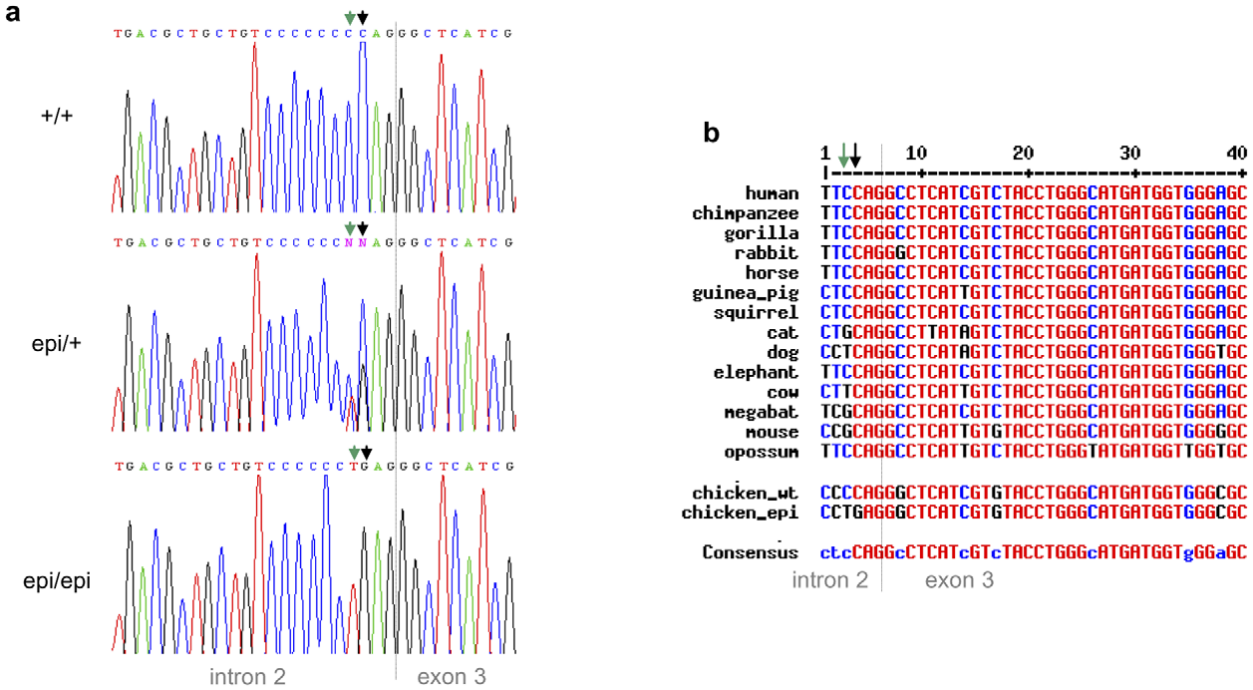
Identification of a dinucleotide mutation that causes reflex epilepsy in the Fepi chicken strain. (a) Sequencing electropherograms of the *SV2A* intron 2 fragment showing the candidate dinucleotide mutation (green and black arrows). Abbreviations: wild-type, CC; heterozygous, CC/TG; epileptic, TG. (b) Multalin [59] sequence comparison between mammals, wild-type chickens and epileptic chickens in the candidate mutation region. Colors: high degree of consensus (red), low degree of consensus (blue) and single change (black). The second C nucleotide (black arrow) is highly conserved across the various species, whereas the first C nucleotide (green arrow) was found to be replaced by a T (in dog and cow) or a G (in cat, mouse and megabat), suggesting that the former is more likely to be responsible for the epileptic phenotype.

Our results indicated that the mutation was likely to be located within the intronic sequence, so we sequenced five long-range PCR products from two heterozygous carrier sires. We identified a dinucleotide mutation in the acceptor site of intron 2 (c581-4CC>TG) that could explain the abnormal splicing observed in the mRNA samples from epileptic chicken brains. To confirm this candidate mutation, the relevant fragment of *SV2A* intron 2 was sequenced from 185 wild-type chickens from 16 different lines, 40 heterozygous carriers, and 145 epileptic chickens. Our results revealed that all of the wild-type individuals carried the CC allele, whereas the carriers were heterozygous (CC/TG) and the epileptics were homozygous for the TG allele (Fig. 3a). We further found that the candidate mutation is located in a region that is conserved across species (Fig. 3b), and further noted that while a C→T mutation was often observed in other species, no other instance of the C→G mutation was found. We thus propose that the C→G mutation is the causative mutation for photosensitive epilepsy in the Fepi chicken. The obtained sequence is available at NCBI (Accession Number JN232407).

### Characterization of the mutation by expression and *in silico* analyses

**Figure 4:**
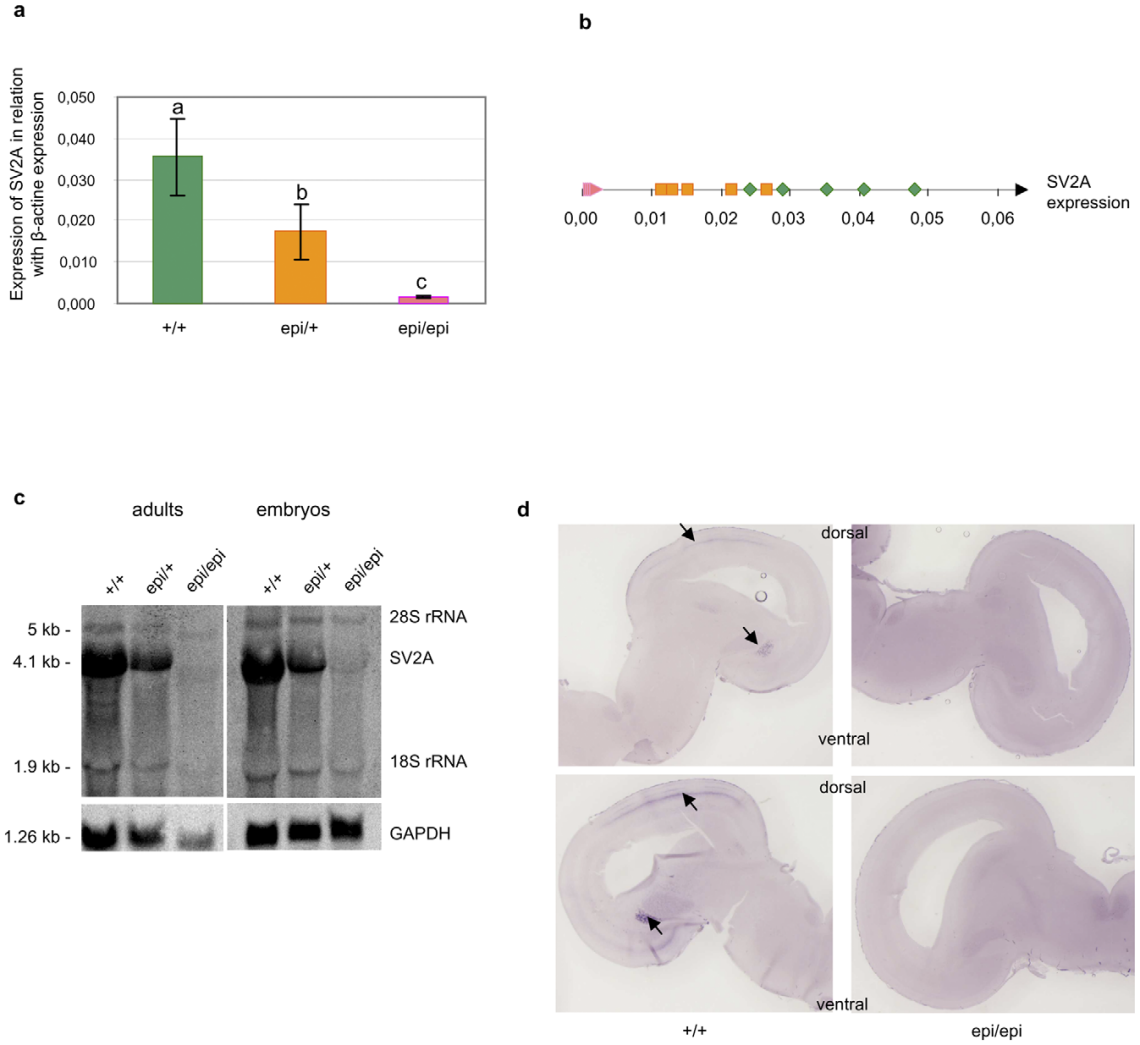
Expression analysis of *SV2A* in the Fepi chicken strain. (a) Real-time relative RT-PCR analysis showing the expression of *SV2A* versus that of β-actin in chicken embryos from the three genotypes (wild-type, carriers and *epi/epi*; n = 5 for each). The average *SV2A* expression was found to be higher in wild-type chickens compared to carriers and epileptic chickens, and in carriers compared to epileptics (p<0.003, Kruskal-Wallis test). (b) The relative expression in each tested embryo is shown in color by genotype: green, wild-type; orange, carrier; and pink, *epi/epi*. (c) Northern blot analysis showing that *SV2A* expression is highest in wild-type embryos and adults. Size estimations are indicated in kb. (d) *In situ* hybridization analysis of brains cross-sections from wild-type and *epi/epi* 17 day-old chicken embryos (E17). *SV2A* is clearly expressed in the mesencephalon of the wild-type embryonic brain (black arrow) but not in *epi/epi* embryos.

The identified abnormal splicing event is fully associated with the epileptic phenotype (Fig. 2a). Interestingly, homozygous *epi* mutants express the wild-type mRNA, and both heterozygous carriers in this study and mice hemizygous for *SV2A* [18] fail to display epilepsy. This suggests that a half dose of the wild-type transcript is sufficient to avoid seizure. Notably, however, *SV2A* knock-out mice experience severe seizures and die by three weeks of age, showing that the total absence of the transcript is lethal [18]. Although the level of normal *SV2A* transcripts is much lower in homozygous *epi* mutants as compared to *epi*/+ chickens, this low level is sufficient to circumvent lethality. Indeed analyses of *SV2A* expression levels by relative real-time PCR showed that there was a genotype-dependent differential expression of the gene (Fig. 4a and b). Heterozygous carriers displayed two-fold lower expression compared to the wild type (0.017+/−0.006 versus 0.035+/−0.009), while epileptic chicken brains showed a very low level of *SV2A* mRNA (0.0019+/−0.0005), i.e., 25-fold lower than that of the wild type and 12.5-fold lower than that of the heterozygous carriers. Consistent with our relative real-time PCR analysis, Northern blot analysis showed a weak signal for the normal 4.1-kb mRNA and no minor band in *epi/epi* chickens (Fig. 4c). *In situ* hybridization analyses performed on brain cross sections confirmed that the level of *SV2A* transcripts was reduced in *epi/epi* versus wild-type chickens (Fig. 4d).

The use of human splicing finder software [21] for *in silico* analysis of the acceptor site (3'ss, splice site) of intron 2 of *SV2A* showed that, consistent with our experimental data, the mutant (TG) allele had significantly lower constitutional 3'ss strength compared to the wild-type (CC) allele (Fig. 4). Furthermore, substitution of the first C nucleotide with a T to create the hypothetical TC allele did not decrease the strength of the constitutional 3'ss, whereas substitution of the second C with a G to create the hypothetical CG allele yielded a result similar to that generated by the candidate mutation, regardless of whether we were using the HSF matrix [21] or the MaxEnt matrix [22] (Fig. S5). This supports the hypothesis that the C→G mutation is the real causative mutation of the *epi* phenotype, resulting in an abnormal splice variant.

The proportion of disease-causing mutations involving splicing defects has been estimated in different studies, ranging from 10 to 60% [23], [24]. Two recent analyses estimate to about one-third the percentage of mutations of this type [25], [26], being involved in a wide range of disorders [27]. Among them, mutations inducing seizure have already been observed, for example in the *ALDH7A1* [28] or *MTHFR* genes [29]. Beyond nucleotide mutations causing abnormal splicing, an increasing number of studies highlight the implication of splicing variations in gene expression regulation. This is manifest for neuronal physiology: several studies underline the importance of alternative splicing in the development of neurological disorders such as epilepsy by affecting different genes, like *GPHN*, encoding the post-synaptic protein gephyrin [30], *SCN1A* (type 1 sodium channels) [31], or the bromodomain-containing *BRD2* gene [32]. The recent analysis of a mouse with a central nervous system-specific deletion of the splicing-regulator *Rbfox1* confirms the importance of splicing regulation in neuronal physiology [33].

### Testing the effect of levetiracetam

The SV2A protein is the binding site for levetiracetam (Keppra®; UCB Pharma S.A). This antiepileptic drug [34] has documented effects on seizures in the photosensitivity model [35]. Our present data suggest that epi/epi chickens survive because they still express low levels of normal SV2A yet not high enough to protect against seizure. If this statement is true we expect that epi/epi chicken will still be sensitive to levetiracetam and that the antiepileptic drug will at least partially rescue the phenotype. We tested the antiepileptic effect of levetiracetam in our chicken model and found that it reduced the number of seizures (odds ratio 0.03, p = 9e-07), delayed the appearance of myoclonus (from 22.8 s to 25.1 s on average, p = 2e-03) and reduced the duration of seizures (from 16.2 s to 10.7 s on average, p = 2e-08) when we compared e*pi/epi* chickens treated with 50, 100 or 200 mg/kg versus those receiving a placebo. We further found that the effect partially persisted for a few days after the injection period. These findings indicate that levetiracetam has an anticonvulsant effect in the chicken model supporting our proposal that a low level of SV2A allows survival but is not sufficient to protect against seizure, or suggesting another pathway of action of this drug. Thus, the sensitivity of the Fepi chicken to levetiracetam reinforces the interest of this model, as other studies have shown lower effects of levetiracetam in mice expressing only one copy of SV2A [36] and in chronic-treated epileptic rats [37] or humans [38], [39]. As previously noted [40], the mechanism of action of *SV2A* and its interaction with levetiracetam have not yet been fully elucidated [41] and the Fepi chicken model could be used for such studies. A recently developed mouse model for epilepsy exhibits a triple knock-out of synapsin genes [42] and shows indeed a relationship between the efficiency of the levetiracetam treatment and the level of SV2A receptor. The Fepi chicken brings a unique opportunity to focus on the role of SV2A alone and to study quantitative effects of SV2A expression in a fully viable vertebrate.

In sum, we herein show that photosensitive epilepsy in the Fepi chicken is associated with an abnormal splicing event affecting the *SV2A* gene, which leads to significantly decreased expression in epileptic (homozygous) chickens. We suggest that the IVS2-4CC>TG substitution in *SV2A* (most likely the IVS2-3C>G) is the causative mutation. Our results support the presence of a gene dosage effect: the quantity of SV2A present in heterozygous animals is sufficient to prevent seizures, while the level present in homozygous animals is not. We thus identified for the first time the molecular basis of a genetic reflex epilepsy, which should pave the way to functional in-depth studies of this monogenic epilepsy model.

## Materials and Methods

### Ethics statement

This study was carried out at INRA (Pôle d'Expérimentation Avicole de Tours, F-37380 Nouzilly, authorization B37-175-1, 2007) in accordance with European Union Guidelines for animal care, under authorization 37-002 delivered to D. Gourichon by the French Ministry of Agriculture. Animal procedure was approved by Departmental Direction of Veterinary Services of Indre-et-Loire.

### Animals

The experimental pedigree, derived from a Fayoumi ancestor, comprised two half-sib families: two heterozygous sires from an epileptic family were each crossed with five or six affected homozygous dams, and yielded a total of 209 offspring. Blood samples were collected from all individuals of the experimental pedigree (born in 2002) and DNA was extracted. Samples were also collected from animals of the subsequent generations (2003: n = 18; 2004: n = 28; 2005: n = 151; 2006: n = 30; 2007: n = 134; 2008: n = 120; 2009: n = 146). The wild-type animals used to confirm the causative mutation came from 16 different lines: two lines differing for coccidiosis resistance (originating from White Leghorn and Fayoumi [43]); two divergent lines selected for meat quality (originating from broiler lines [44]); two divergent lines selected for their growth curves (originating from broiler lines [45]); two divergent lines selected for residual feed intake (originating from Rhode Island Red [46]); three divergent lines selected for salmonella resistance [originating from White Leghorn; these were experimental inbred lines from the USDA Avian Disease and Oncology Laboratory (East Lansing, MI) and were provided by the Institute for Animal Health (IAH; Compton, UK)]; the East Lansing backcross (originating from Red Jungle Fowl and White Leghorn [47]); one inbred line (originating from Leghorn [48]); one commercial broiler line; one experimental Naked Neck line (originating from a laying strain [49]) and one experimental line selected for resistance to Rous sarcomas (originating from White Leghorn [50]).

### Phenotyping

All animals in the experimental population were tested twice for the photosensitive GRS (Genetic Reflex Seizure), with the exception of animals born in 2005, which were tested only once. The first phenotyping test took place at birth and the second one at one week (2009), three weeks (2002), six weeks (2007 and 2008) or eight weeks (2003–2006) of age. Tests consisted of intermittent light stimulation at 14 flashes per second, which is the most effective epileptogenic frequency for Fepi chickens [51]. Animals were classified as epileptic if they displayed a seizure during at least one of the two tests.

### Genotyping

PCR amplifications and genotyping of markers 100A3M13, SEQ1285 and GCT1888 (Table S1) were performed through SSCP (Single Strand Conformation Polymorphism) or PCR-RFLP (Restriction Fragment Length Polymorphism) as previously described [17]. Genotyping of markers GCT2123 and GCT2044 (Table S1) was performed via SSCP (Single-Strand Conformation Polymorphism) analysis on an ABI 3100 sequencer (Applied Biosystems), as described in Applied Biosystems Publication 116AP01-02. Marker GCT2272 (Table S1) was genotyped by direct sequencing using an ABI 3730 sequencer (Applied Biosystems). Linkage analysis was performed using the CriMap version 2.4 software [52]. The “build” option was used to order markers within the linkage group, while the “flips” option was used to confirm the order of the markers.

### Sequencing

#### Sanger technology

Twenty-four PCR fragments were amplified on an ABI 9700 thermocycler (Applied Biosystems) from cDNA generated from two wild-type chickens, two carriers and two epileptic animals. Amplifications were performed using a GC-rich PCR system (Roche Applied Science) and primer pairs GCT1967U-GCT1694L, GCT1964U-GCT2146L, GCT2146U-GCT2044L and GCT2151U-GCT2254L (Table S1). The fragments were purified using 0.5 U of SAP (Shrimp Alkaline Phosphatase, Promega) and 0.5 U of exonuclease I (NE Biolabs), sequenced using a Big Dye Terminator v3.1 Kit (Applied Biosystems), and analyzed on ABI 3730 or ABI 3100 sequencers (Applied Biosystems).

#### 454 Technology

Five long-range PCR fragments were amplified on an ABI 9700 thermocycler (Applied Biosystems) from the two heterozygous sires, using the Long PCR Enzyme Mix (Fermentas) and primer pairs SNP36U-GCT1964L, GCT1964U-GCT2244L, GCT2146U-GCT2147L, GCT2148U-GCT2152L and GCT2152U-GCT2245L (Table S1). The resulting fragments were purified and pooled all together at equal concentrations. These samples were then sequenced using the Roche 454 Life Sciences Genome Sequencer FLX (454 Life Science, Roche), following the manufacturer's instructions with the following kits (454 Life Science, Roche): a shotgun library was prepared with 1 µg genomic DNA using the Titanium General Library Preparation Kit. Nebulized, purified, and adaptor-linked DNA fragments were amplified using the GS FLX Titanium LV emPCR Kit, and sequencing on the FLX Genome Sequencer was performed using the GS FLX Sequencing Kit, Titanium Reagents XLR70. A total of 245,625 reads were obtained, with an average length of 340 bases. Contig building was performed using the AMOS comparative assembler [53].

### RNA extraction

Total RNA was extracted from adult and embryonic (E17) chicken brains according to the technique described by Le Meur et al. [54], with slight modification [55].

### Relative real-time PCR

The utilized cDNA were generated in 20-µl reaction volumes containing 2 µg of total RNA, 1 µM of polydT primer (Roche), 200 U of Superscript II reverse transcriptase (Invitrogen), 40 U of RNasin (Promega) and 0.04 µM of dNTP. *SV2A* expression was analyzed using the relative expression method described by Drouilhet et al. [56], using the primers listed in Table S1. The level of ACTB (β-actin) gene expression was used to normalize the amount of each investigated transcript. PCR was performed in 10-µl reaction volumes using LightCycler®480 SYBR Green I Master (Roche) and 3 µM each of the forward and reverse primers. PCR was performed in a LightCycler 480 instrument (Roche). All samples were analyzed in duplicate.

### Northern blot analysis

RNA was electrophoretically separated on a denaturing formaldehyde agarose gel, transferred to a nylon membrane (Millipore) and immobilized by UV irradiation. The utilized ^33^P-labeled probes were generated from two PCR products representing the *SV2A* cDNA and one PCR product representing GAPDH (reference gene) using the Prime-a-gene protocol (Promega), and purified on microspin G50 columns (GE Healthcare). For Northern blotting, the hybridization buffer was composed of 5× SSC, 5× Denhardt's solution and 0.5% SDS, and the washing buffer was composed of 0.2× SSC and 0.1% SDS. The membranes were pre-hybridized for 2 h at 65°C with hybridization buffer and 10 µg/ml sonicated salmon sperm DNA, and hybridized overnight at 65°C with the relevant ^33^P-labeled probe (2 000 000 CPM/ml of hybridization buffer). The membranes were then washed twice (15 minutes and 5 minutes, respectively) with washing buffer, and exposed to screens for 24 h (GAPDH probe) or 7 days (SV2A probes). The screens were then scanned with a Fujibas 5000 instrument (Fuji), and results were analyzed using the ArrayGauge V1.3.S software (Fuji).

### *In situ* hybridization analysis

*In situ* hybridization was performed as previously described [57] on Vibratome-cut (VT 1000S Vibrating Blade Microtome, Leica) 50-µm brain cross sections obtained from E17 embryos. Nonradioactive RNA *in situ* hybridizations were performed using digoxigenin-UTP (Roche) and a labeled RNA probe corresponding to a 680-bp PCR fragment from the *SV2A* cDNA.

### Testing the effect of levetiracetam

The antiepileptic effect of levetiracetam (Keppra®, UCB Pharma) was tested on 71 three to five month-old Fepi chickens (identified as having ILS_induced seizures at birth). All chickens were exposed to ILS once a day for 21 days. On day one, all showed ILS-induced seizures (baseline control). Over the following 10 days, all animals received one intraperitoneal injection per day as follows: 18 animals received 200 mg/kg of levetiracetam; 17 received 100 mg/kg; 18 received 50 mg/kg; and 18 control animals received a placebo injection. Each treatment category (200, 100, 50, 0) included equal proportions of males and females. No animal was treated during the last 10 days of ILS testing. The influence of levetiracetam treatment on the stimulus-locked myoclonus and the generalized convulsions of the photosensitive GRS was evaluated using generalized linear models implemented in the glm function of the R software, version 2.9.0 [58]. The number of seizures for each animal over a given phase of the experiment (during treatment and following treatment) was modeled using a binomial distribution, and linked to factors using the logit function. The delay before seizure initiation, the duration of the myoclonus, and the duration of the convulsions were modeled using gamma distributions and linked to the factors using the identity function. Sex was included as an additive co-factor in the analyses, but no significant difference was found.

## Supporting Information

Figure S1 **The Fepi mapping pedigree.** At generation 0 (2001), two heterozygous sires from an epileptic family were each crossed with five or six affected homozygous dams, giving rise to a total of 209 offspring born in 2002. Successive crosses were performed each year thereafter, for seven years, in an attempt to obtain offspring with crossover events close to the epi mutation.
(DOC)

Figure S2 **Available chicken genomic sequences representing the genetic interval containing the epi mutation.** The epi mutation was localized to a region of GGA25 that was only poorly covered by the chicken genome assembly, where just over 1.5 Mb of gapped sequence represented this chromosome, which has an estimated size of 11.4 Mb. Alignment of our framework genetic map with the sequence assembly of GGA25 showed that only one side of both our initial GCT1888-SEQ1285 interval and our refined GCT1888-GCT2123 interval was present in the assembly, and that there were many gaps representing missing sequences. GCT1888 is located on GGA25, while GCT2123 had been designated to chrUn, which contains sequences that have not yet been attributed to a specific chicken chromosome. (From the UCSC genome browser: http://genome.ucsc.edu/cgi-bin/hgGateway.)
(DOC)

Figure S3 **Identification of additional sequences by comparison to the human genome.** Sequence searching with our 6.6-cM mapped genetic interval (markers GCT1888 and SEQ1285, black arrows) identified a similar region in the human genome, located on HSA1q21.2. This region represents 3.94 Mb of HSA1, most of which does not correspond to GGA25 (in gray and boxed in the chicken Alignment Net), but rather to GGA8 (orange) or GGA1 (brown) and other chicken chromosomes. This restricted the candidate region to sequences near GCT1888, which contained a single candidate gene: SV2A. (From the UCSC genome browser http://genome.ucsc.edu/cgi-bin/hgGateway.)
(DOC)

Figure S4 **Finding additional sequence information for the chicken SV2A gene.** (a) Chicken SV2B (GGA10, yellow) and a portion of SV2C (GGAZ, black) aligned with human SV2A, but only a small portion of the sequence was available for chicken SV2A (GGA25, gray). (b) When we observed the alignment of available chicken sequence against that from the mouse genome assembly, however, we were able to identify additional chicken sequences from chrUn_random (contigs 1773.1 and 1773.2, March 2006 assembly) that aligned with the mouse SV2A gene. This additional sequence information was used to develop new SNP markers.
(DOC)

Figure S5 **Analysis of the splicing acceptor site using the Human Splicing Finder software.** The first graph (a) shows the acceptor site strength for each putative allele, as determined using the HSF matrix, which considers the last 12 nucleotides of the intron and the first nucleotide of the following exon. Below the threshold value of 80, the acceptor site is considered non-existent. The second graph (b) shows the acceptor site strength calculated based on the MaxEnt matrix, which considers the last 20 nucleotides of the intron and the first three of the following exon. Below a threshold of 0, the acceptor site is considered non-existent. CC: Wild type allele; TC: Hypothetical recombinant haplotype; CG: Hypothetical recombinant haplotype; TG: Mutant allele (epi).
(DOC)

Table S1 **Markers used for genetic and expression analyses.** Positions (Start and End columns) are given in Mb relative to WUGSC 2.1 chicken sequence assembly (UCSC) or, when available, relative to the SV2A gene structure. Markers used for genetic analysis are labeled with *, primers marked “a” are extended with M13 probe (5′-GTTTTCCCAGTCACGACGTTG-3′) and primer marked “b” is extented with rM13 probe (5′-AGGAAACAGCTATGACCATGAT-3′). Markers used for relative real time PCR analysis are labeled with #.
(DOC)
