# Supplementary material for: Epilepsy Caused by an Abnormal Alternative Splicing with Dosage Effect of the SV2A Gene in a Chicken Model
Source: PLoS One. 2011 Oct 27;6(10):e26932. doi: 10.1371/journal.pone.0026932 (PMC3203167; doi:10.1371/journal.pone.0026932)
Supplement: Figure S1 — The Fepi mapping pedigree. At generation 0 (2001), two heterozygous sires from an epileptic family were each crossed with five or six affected homozygous dams, giving rise to a total of 209 offspring born in 2002. Successive crosses were performed each year thereafter, for seven years, in an attempt to obtain offspring with crossover events close to the epi mutation. (DOC) [file pone.0026932.s001.doc]

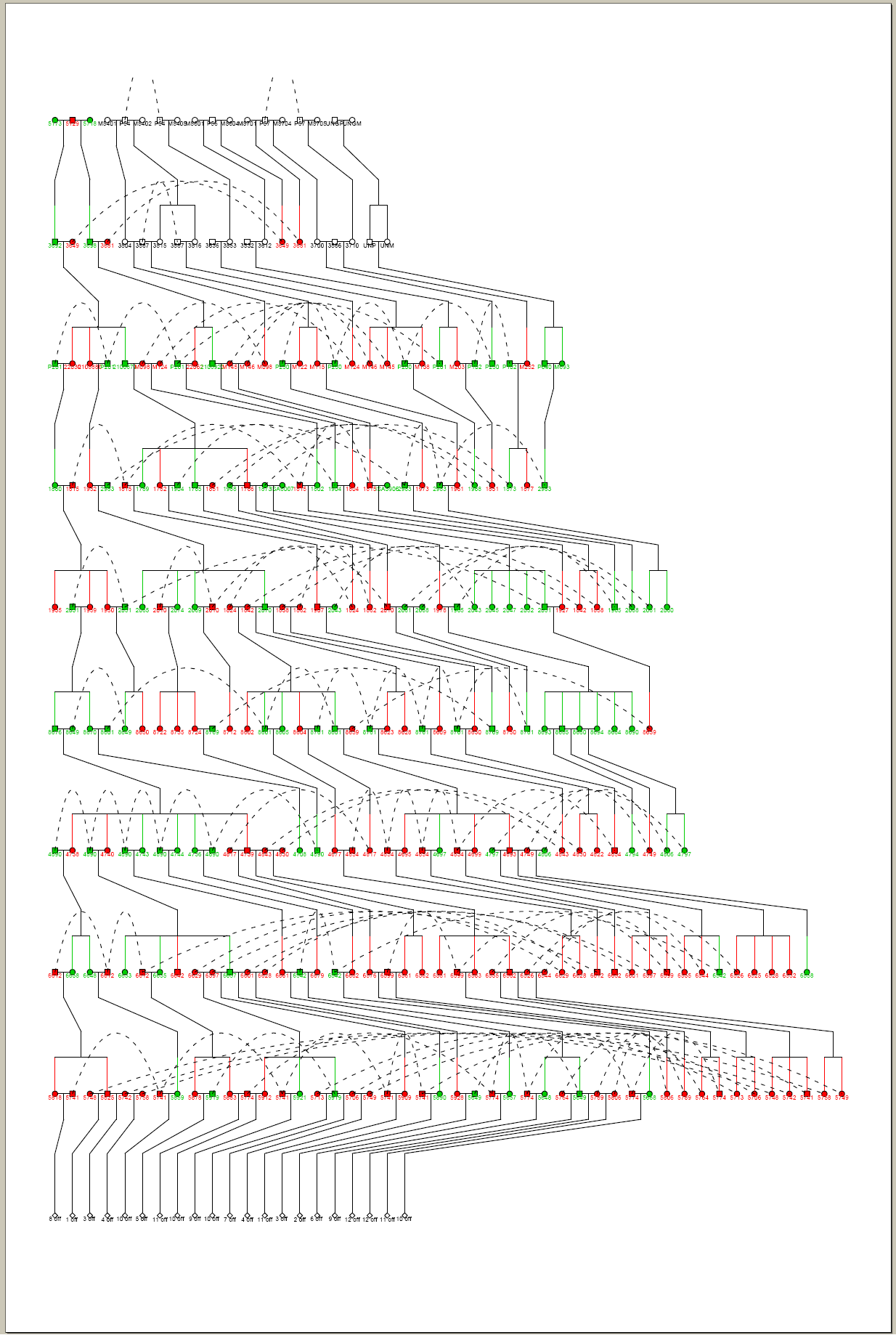


2000

Generation -1

2001

Generation 0

2005

Generation 4

2002

Generation 1

2003

Generation 2

2004

Generation 3

2006

Generation 5

2009

Generation 8

2008

Generation 7

2007

Generation 6

**Figure S1. The Fepi mapping pedigree**

At generation 0 (2001), two heterozygous sires from an epileptic family were each crossed with five or six affected homozygous dams, giving rise to a total of 209 offspring born in 2002. Successive crosses were performed each year thereafter, for seven years, in an attempt to obtain offspring with crossover events close to the *epi* mutation.
