# Supplementary material for: Epilepsy Caused by an Abnormal Alternative Splicing with Dosage Effect of the SV2A Gene in a Chicken Model
Source: PLoS One. 2011 Oct 27;6(10):e26932. doi: 10.1371/journal.pone.0026932 (PMC3203167; doi:10.1371/journal.pone.0026932)
Supplement: Figure S2 — Available chicken genomic sequences representing the genetic interval containing the epi mutation. The epi mutation was localized to a region of GGA25 that was only poorly covered by the chicken genome assembly, where just over 1.5 Mb of gapped sequence represented this chromosome, which has an estimated size of 11.4 Mb. Alignment of our framework genetic map with the sequence assembly of GGA25 showed that only one side of both our initial GCT1888-SEQ1285 interval and our refined GCT1888-GCT2123 interval was present in the assembly, and that there were many gaps representing missing sequences. GCT1888 is located on GGA25, while GCT2123 had been designated to chrUn, which contains sequences that have not yet been attributed to a specific chicken chromosome. (From the UCSC genome browser: http://genome.ucsc.edu/cgi-bin/hgGateway.) (DOC) [file pone.0026932.s002.doc]

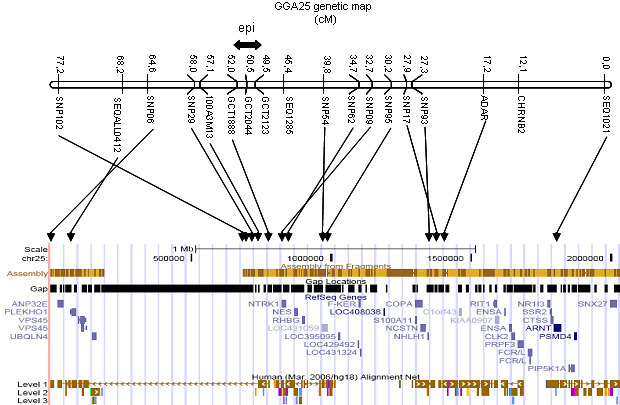


**Figure S2.** **Available chicken genomic sequences representing the genetic interval containing the *epi* mutation**

The *epi* mutation was localized to a region of GGA25 that was only poorly covered by the chicken genome assembly, where just over 1.5 Mb of gapped sequence represented this chromosome, which has an estimated size of 11.4 Mb. Alignment of our framework genetic map with the sequence assembly of GGA25 showed that only one side of both our initial GCT1888-SEQ1285 interval and our refined GCT1888-GCT2123 interval was present in the assembly, and that there were many gaps representing missing sequences. GCT1888 is located on GGA25, while GCT2123 had been designated to chrUn, which contains sequences that have not yet been attributed to a specific chicken chromosome. (From the UCSC genome browser: <http://genome.ucsc.edu/cgi-bin/hgGateway>.)
