# Supplementary material for: Epilepsy Caused by an Abnormal Alternative Splicing with Dosage Effect of the SV2A Gene in a Chicken Model
Source: PLoS One. 2011 Oct 27;6(10):e26932. doi: 10.1371/journal.pone.0026932 (PMC3203167; doi:10.1371/journal.pone.0026932)
Supplement: Figure S3 — Identification of additional sequences by comparison to the human genome. Sequence searching with our 6.6-cM mapped genetic interval (markers GCT1888 and SEQ1285, black arrows) identified a similar region in the human genome, located on HSA1q21.2. This region represents 3.94 Mb of HSA1, most of which does not correspond to GGA25 (in gray and boxed in the chicken Alignment Net), but rather to GGA8 (orange) or GGA1 (brown) and other chicken chromosomes. This restricted the candidate region to sequences near GCT1888, which contained a single candidate gene: SV2A. (From the UCSC genome browser http://genome.ucsc.edu/cgi-bin/hgGateway.) (DOC) [file pone.0026932.s003.doc]

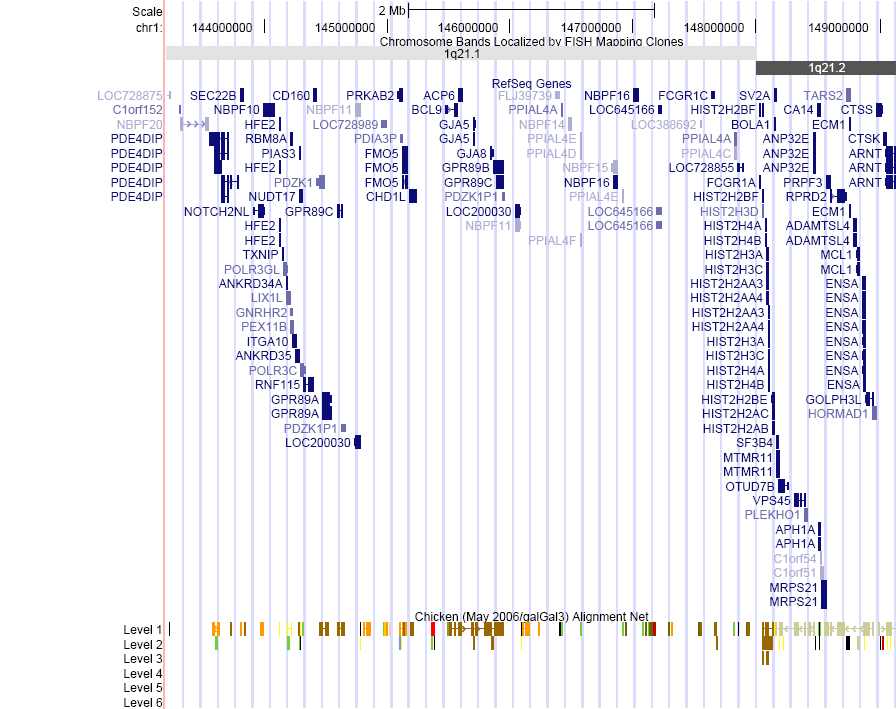


**SEQ1285**

**GCT1888**

**GGA25**

**Figure S3.**  **Identification of additional sequences by comparison to the human genome** Sequence searching with our 6.6-cM mapped genetic interval (markers GCT1888 and SEQ1285, black arrows) identified a similar region in the human genome, located on HSA1q21.2. This region represents 3.94 Mb of HSA1, most of which does not correspond to GGA25 (in gray and boxed in the chicken Alignment Net), but rather to GGA8 (orange) or GGA1 (brown) and other chicken chromosomes. This restricted the candidate region to sequences near GCT1888, which contained a single candidate gene: *SV2A*. (From the UCSC genome browser <http://genome.ucsc.edu/cgi-bin/hgGateway>.)
