# Supplementary material for: Epilepsy Caused by an Abnormal Alternative Splicing with Dosage Effect of the SV2A Gene in a Chicken Model
Source: PLoS One. 2011 Oct 27;6(10):e26932. doi: 10.1371/journal.pone.0026932 (PMC3203167; doi:10.1371/journal.pone.0026932)
Supplement: Figure S4 — Finding additional sequence information for the chicken SV2A gene. (a) Chicken SV2B (GGA10, yellow) and a portion of SV2C (GGAZ, black) aligned with human SV2A, but only a small portion of the sequence was available for chicken SV2A (GGA25, gray). (b) When we observed the alignment of available chicken sequence against that from the mouse genome assembly, however, we were able to identify additional chicken sequences from chrUn_random (contigs 1773.1 and 1773.2, March 2006 assembly) that aligned with the mouse SV2A gene. This additional sequence information was used to develop new SNP markers. (DOC) [file pone.0026932.s004.doc]

**a: alignment to human *SV2A***

**b: alignment to mouse *SV2A***


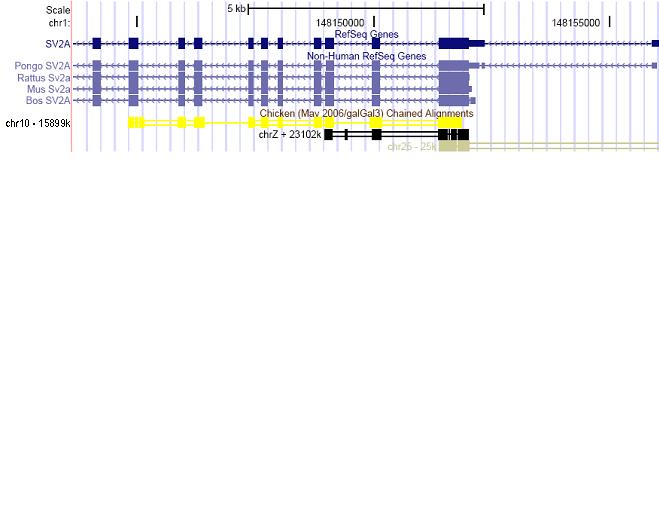

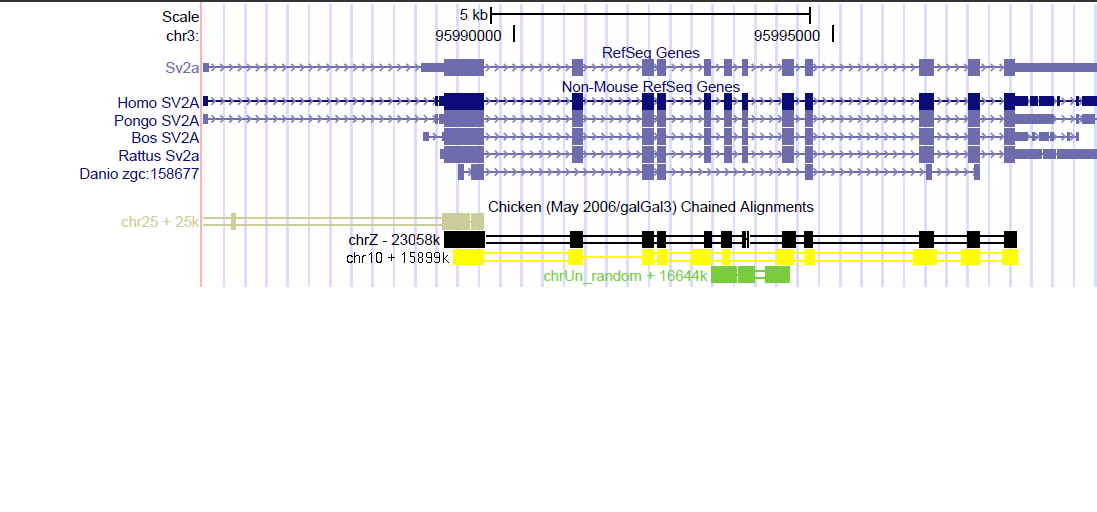


**Figure** **S4.**  **Finding additional sequence information for the chicken *SV2A* gene**

(a) Chicken *SV2B* (GGA10, yellow) and a portion of *SV2C* (GGAZ, black) aligned with human *SV2A*, but only a small portion of the sequence was available for chicken *SV2A* (GGA25, gray). (b): When we observed the alignment of available chicken sequence against that from the mouse genome assembly, however, we were able to identify additional chicken sequences from chrUn_random (contigs 1773.1 and 1773.2, March 2006 assembly) that aligned with the mouse *SV2A* gene. This additional sequence information was used to develop new SNP markers.
