# Supplementary material for: Epilepsy Caused by an Abnormal Alternative Splicing with Dosage Effect of the SV2A Gene in a Chicken Model
Source: PLoS One. 2011 Oct 27;6(10):e26932. doi: 10.1371/journal.pone.0026932 (PMC3203167; doi:10.1371/journal.pone.0026932)
Supplement: Figure S5 — Analysis of the splicing acceptor site using the Human Splicing Finder software. The first graph (a) shows the acceptor site strength for each putative allele, as determined using the HSF matrix, which considers the last 12 nucleotides of the intron and the first nucleotide of the following exon. Below the threshold value of 80, the acceptor site is considered non-existent. The second graph (b) shows the acceptor site strength calculated based on the MaxEnt matrix, which considers the last 20 nucleotides of the intron and the first three of the following exon. Below a threshold of 0, the acceptor site is considered non-existent. CC: Wild type allele; TC: Hypothetical recombinant haplotype; CG: Hypothetical recombinant haplotype; TG: Mutant allele (epi). (DOC) [file pone.0026932.s005.doc]

76

78

80

82

84

86

88

90

92

94

96

**a**

**b**

0

2

4

6

8

10

12

CC

TC

TG

CG

CC

TC

TG

CG

**Figure S5. Analysis of the splicing acceptor site using the Human Splicing Finder software**

The first graph (a) shows the acceptor site strength for each putative allele, as determined using the HSF matrix, which considers the last 12 nucleotides of the intron and the first nucleotide of the following exon. Below the threshold value of 80, the acceptor site is considered non-existent. The second graph (b) shows the acceptor site strength calculated based on the MaxEnt matrix, which considers the last 20 nucleotides of the intron and the first three of the following exon. Below a threshold of 0, the acceptor site is considered non-existent.

CC: Wild type allele; TC: Hypothetical recombinant haplotype; CG: Hypothetical recombinant haplotype; TG: Mutant allele (*epi*).
