# Supplementary material for: Epilepsy Caused by an Abnormal Alternative Splicing with Dosage Effect of the SV2A Gene in a Chicken Model
Source: PLoS One. 2011 Oct 27;6(10):e26932. doi: 10.1371/journal.pone.0026932 (PMC3203167; doi:10.1371/journal.pone.0026932)
Supplement: Table S1 — Markers used for genetic and expression analyses. Positions (Start and End columns) are given in Mb relative to WUGSC 2.1 chicken sequence assembly (UCSC) or, when available, relative to the SV2A gene structure. Markers used for genetic analysis are labeled with *, primers marked “a” are extended with M13 probe (5′-GTTTTCCCAGTCACGACGTTG-3′) and primer marked “b” is extented with rM13 probe (5′-AGGAAACAGCTATGACCATGAT-3′). Markers used for relative real time PCR analysis are labeled with #. (DOC) [file pone.0026932.s006.doc]

**Table S1: Markers used for genetic and expression analyses.**

Positions (Start and End columns) are given in Mb relative to WUGSC 2.1 chicken sequence assembly (UCSC) or, when available, relative to the SV2A gene structure. Markers used for genetic analysis are labeled with *, primers marked "a" are extended with M13 probe ( 5'-GTTTTCCCAGTCACGACGTTG-3') and primer marked "b" is extented with rM13 probe (5'-AGGAAACAGCTATGACCATGAT-3'). Markers used for relative real time PCR analysis are labeled with #.

| Marker name | Marker localisation | Start | End | Primer name | Primer Sequence (5'-3') |
| --- | --- | --- | --- | --- | --- |
| 100A3M13^*^ | GGA25 | 739,414 | 739,615 | 100A3M13U | CCTCATTTAATTTCATCACTGG |
|  |  |  |  | 100A3M13L^a^ | TCTCCGAGCACAGGACTTC |
| GCT1888^*^ | GGA25 | 776,241 | 776,574 | GCT1888U | AATCCTCCAGTTGCCATCC |
|  |  |  |  | GCT2003L | GTGCTTCTTCACCTCCTTGG |
| GCT2044^*^ | unknown | 16644846 SV2A exon7 | 16645120 SV2A exon8 | GCT2044U | CCCACATCAAGACCATCAAG |
|  |  |  |  | GCT2044L^b^ | ACACGGCCATCATCATCAG |
| GCT2123^*^ | no hit | / SV2A intron8 | / SV2A intron8 | GCT2123U^a^ | GTCGTCCAGTTGCCATTATC |
|  |  |  |  | GCT2123L | GCTGACAGTGTTGGGACAAG |
| GCT2272^*^ | no hit | / SV2A intron2 | / SV2A exon3 | GCT2272U | TGTCCCTGCTACCCGGG |
|  |  |  |  | GCT2272L^a^ | CAGAACAGGAAGGTGCCGTAG |
| SEQ1285^*^ | unknown | 7,142,815 | 7,143,151 | SEQ1285U^a^ | GAGGTGCCGAAATTGGACA |
|  |  |  |  | SEQ1285L | TCATCGGTACTCACAGCG |
| GCT1964 | GGA25 | 776771 SV2A exon2 | / SV2A exon4 | GCT1964U | TCTACGAGGGCGAGTACCAG |
|  |  |  |  | GCT2146L | CCAACTCAGGTGCTCAC |
| GCT1967 | GGA25 | 776502 SV2A exon2 | 776977 SV2A exon2 | GCT1967U | GGATGAGAGCTTCCGAGAC |
|  |  |  |  | GCT1964L | GTCCCAGCACGAAGTAGAGG |
| GCT2146 | no hit | / SV2A exon3 | / SV2A exon8 | GCT2146U | GTACCTGGGCATGATG |
|  |  |  |  | GCT2044L | ACACGGCCATCATCATCAG |
| GCT2147 | no hit | / SV2A exon3 | / SV2A exon6 | GCT2146U | GTACCTGGGCATGATG |
|  |  |  |  | GCT2147L | CCTTGGCCCTCATGTTG |
| GCT2148 | no hit | / SV2A exon5 | / SV2A exon9 | GCT2148U | GCTGGAGCTTCCAGATGG |
|  |  |  |  | GCT2152L | CGTTGAAGTACTCCCCG |
| GCT2151 | no hit | / SV2A intron5 | / SV2A | GCT2151U | CAGGCTCTGCCCTGCAG |
|  |  |  |  | GCT2254L | TGGCAGAGGGACTGAGACC |
| GCT2152 | no hit | / SV2A intron8 | / SV2A intron10 | GCT2152U | TACTACGGGCTGACGGT |
|  |  |  |  | GCT2245L | GTTCCAGGAGGCGATGC |
| GCT2244 | GGA25/no hit | 776771 SV2A exon2 | / SV2A exon3 | GCT1964U | TCTACGAGGGCGAGTACCAG |
|  |  |  |  | GCT2244L | TGACGGAGAGGGAGATGAG |
| SNP36 | GGA25 | 773,039 | 776977 SV2A exon2 | SNP36U | AATGGGAGCTCAATTCTTTC |
|  |  |  |  | GCT1964L | GTCCCAGCACGAAGTAGAGG |
| GCT2258# | no hit | / SV2A exons8/9 | / SV2A exon9 | GCT2258U | TGTGGTTCACCATGAGCTTCAGTTAC |
|  |  |  |  | GCT2258L | AACAGTTTGGTGCGTGAGGC |
| GCT2269 | no hit | / SV2A exon3 | / SV2A exon4 | GCT2269U | TCCAGGGCTACGGCACC |
|  |  |  |  | GCT2269L | CGCCAGGAACTTGGAGAAGTAGG |
| GCT2271# (βactin) | unknown | 51,462,374 | 51,462,474 | GCT2271U | TCGCACCACAGGCATCGTTA |
|  |  |  |  | GCT2271L | GGTCCAGACGCAGGATGGC |
